# The influence of distance and quality of care on place of delivery in rural Ghana

**DOI:** 10.1038/srep30291

**Published:** 2016-08-10

**Authors:** Robin C. Nesbitt, Terhi J. Lohela, Seyi Soremekun, Linda Vesel, Alexander Manu, Eunice Okyere, Chris Grundy, Seeba Amenga-Etego, Seth Owusu-Agyei, Betty R. Kirkwood, Sabine Gabrysch

**Affiliations:** 1 Epidemiology and Biostatistics Unit, Institute of Public Health, Heidelberg University, Heidelberg, Germany; 2 Department of Public Health, University of Helsinki, Helsinki, Finland; 3 Maternal & Child Health Intervention Research Group, Faculty of Epidemiology and Population Health, London School of Hygiene and Tropical Medicine, London, UK; 4 Innovations for Maternal, Newborn and Child Health Initiative, Concern Worldwide US, New York, USA; 5 Kintampo Health Research Center, Ghana Health Service, Kintampo, Ghana; 6 Department of Public Health, Flinders University, Adelaide, South Australia; 7 Department for Disease Control, Faculty of Infectious and Tropical Diseases, London School of Hygiene and Tropical Medicine, London, UK

## Abstract

Facility delivery is an important aspect of the strategy to reduce maternal and newborn mortality. Geographic access to care is a strong determinant of facility delivery, but few studies have simultaneously considered the influence of facility quality, with inconsistent findings. In rural Brong Ahafo region in Ghana, we combined surveillance data on 11,274 deliveries with quality of care data from all 64 delivery facilities in the study area. We used multivariable multilevel logistic regression to assess the influence of distance and several quality dimensions on place of delivery. Women lived a median of 3.3 km from the closest delivery facility, and 58% delivered in a facility. The probability of facility delivery ranged from 68% among women living 1 km from their closest facility to 22% among those living 25 km away, adjusted for confounders. Measured quality of care at the closest facility was not associated with use, except that facility delivery was lower when the closest facility provided substandard care on the EmOC dimension. These results do not imply, however, that we should increase geographic accessibility of care without improving facility quality. While this may be successful in increasing facility deliveries, such care cannot be expected to reduce maternal and neonatal mortality.

Skilled attendance at birth is recommended to reduce the burden of maternal and perinatal mortality and morbidity^1^. In contexts where distances are long or transport connections are weak, delivery in a health facility is a critical component of the strategy to improve maternal and newborn survival^2^,^3^,^4^.

In Ghana, more women delivered in a facility than at home for the first time on a national level in 2008^5^. A “shift in norms towards embracing a biomedical model of delivery care in the country” has been described, and is reportedly driven by a belief in health professionals’ ability to mitigate the dangers of delivery complications^5^. Despite this, Millennium Development Goal 5 was not achieved in Ghana; the 2014 maternal mortality ratio of 380 deaths per 100 000 live births was still far from the goal of 190 by 2015^6^. This could be due to low quality of obstetric care in facilities in Ghana^7^ and also persistent inequalities in access to and use of delivery care^8^.

It has been shown that geographic accessibility strongly influences use of facilities for delivery in many low- and middle-income countries (LMIC)^9^,^10^,^11^ including Ghana^12^,^13^. Travel time or distance to the closest health facility is often used as a measure of access to delivery care, and has consistently been shown to be a significant barrier to use, however, the quality of care offered at the closest facility has not. Facility quality of care has been highlighted as an important issue for care-seeking behavior since the seminal “three phases of delay” framework^14^, and perceived quality of care at the delivery facility is frequently identified as an important determinant of facility delivery in qualitative studies, however, quantitative studies have not consistently found evidence to support this link^15^. For example, while a higher level of emergency obstetric care (EmOC) provision at the closest facility was found to increase facility delivery in Zambia^9^,^10^, no effect of facility capacity on facility delivery was found in Malawi^10^ and Ghana^12^. Possible explanations for this lack of effect include inadequate evaluation of quality in facilities and insufficient variation in facility quality within study areas. Considering a recent proposal to expand quality evaluations beyond emergency obstetric care^16^, it might also be relevant to study the influence of other quality dimensions than EmOC on place of delivery.

With this study, we aim to evaluate the influence of distance and quality of care on place of delivery in the Brong Ahafo region in Ghana, considering different quality dimensions: routine delivery and postnatal care, emergency obstetric care and emergency neonatal care.

## Methods

This study is a secondary data analysis of the Newhints cluster-randomized trial from November 2008 to December 2009 on the impact of home visits by community-based surveillance volunteers on neonatal mortality^17^. In 2009, the study area comprised seven districts in the Brong Ahafo region of Ghana, a predominantly rural area with approximately 600,000 residents, of which over 100,000 are women of reproductive age^17^,^18^. Newhints surveillance entailed monthly home visits by resident field workers to all women of reproductive age and women were enrolled in the trial once they became pregnant. Information was collected on socioeconomic characteristics, obstetric history and pregnancy outcome for all women. The neonatal mortality rate in the study area was 31 per 1000 live births^18^, and the national maternal mortality ratio was estimated at 350 per 100,000 live births in 2008^19^. In the same year, Ghanaian national health insurance was made free for all pregnant women, covering all costs associated with pregnancy and delivery, although informal costs may persist^20^.

In 2010, we conducted a health facility assessment (HFA) at all 86 health facilities in the study area. We interviewed the staff member most qualified in maternity care about provision of essential interventions (signal functions) and on staffing, checked competence using clinical vignettes and verified availability of drugs and equipment (tracer items). Quality of routine delivery care, emergency obstetric care (EmOC) and emergency newborn care (EmNC) were each categorized into five levels, combining reported performance of signal functions, tracer items, and minimum requirements on numbers of skilled staff employed at the facility^7^. The health facility assessment identified 64 facilities offering delivery care in the study area: 11 hospitals, 11 maternity homes, 34 health centers and 8 clinics^7^. The facility type “clinics” comprises clinics, ‘community-based health planning and service’ (CHPS) compounds^21^ and health posts. More than half of the 64 facilities (53%, n = 34) were found to provide “good quality” routine care (defined as facilities classified as high or highest on the quality assessment), while less than 20% (n = 12) provided basic or comprehensive emergency obstetric care (BEmOC or CEmOC), and only 8% (n = 5) provided basic or comprehensive emergency newborn care (BEmNC or CEmNC, Table 1).

A geospatial database of the study area was created in ArcMap (ESRI California) mapping all health facilities, roads and villages where pregnant women lived. Distances between village centroids and health facilities were calculated using several different methods, including straight-line distance, road network distance and raster least cost paths, which incorporated topographical barriers^22^. Straight-line distance from the woman’s village to the closest delivery facility proved to be an adequate proxy for potential spatial access to delivery care in this context^22^; therefore, the average village-level distance to the closest health facility was used for all women in the same village in this analysis.

Analyses were conducted in Stata version 12.0, using multilevel logistic regression (*xtmelogit* command), with the lowest level of analysis being the delivery, counting multiple births (twins and triplets) as one delivery, and random intercepts at the village level. Health facility catchment area was considered as an alternative second level, but results were similar and village level accounted for more of the variation in facility use between women. The exposures of interest were straight-line distance to closest facility, facility type and quality of care; the outcome of interest was delivery in a health facility of any type (hospital, clinic, health center, or maternity home). We included the following potential confounders in multivariable models: age, religion, marital status, parity, ethnicity, occupation, wealth quintile, education, multiple birth, previous stillbirth, health insurance and Newhints intervention vs. control group.

Distance to the closest facility was modelled both as a categorical and as a continuous variable using a square-root transformation to approximate a linear association with the log-odds of facility delivery (linearity was checked using lowess plots and fractional polynomials). Health facility type and quality of care were evaluated by adding a categorical variable for the type or quality of care offered at the closest facility to the models including distance as a continuous variable. We also calculated marginal predicted probabilities of facility delivery using the *margins* and associated *marginsplot* post-estimation commands in Stata.

Distance and quality were also modelled in several alternative ways (see supplementary files): distance to the closest facility of a certain quality level (using categorization as described above) and the highest quality facility within a certain distance. Furthermore, two alternative quality measures were evaluated: a simple score counting one point per signal function, per doctor conducting cesarean sections (up to 3) and per health professional (up to 3) at each facility (total maximum 32 points), and health worker competence as evaluated with two clinical vignettes (total maximum 20 points, for details see^23^).

### Ethics

This study uses data collected for the Newhints trial, which was approved by the ethics committees of the Ghana Health Service, Kintampo Health Research Center and the London School of Hygiene and Tropical Medicine (LSHTM) (trial registration number http://ClinicalTrials.gov: NCT00623337)^18^. The additional analyses were approved by these committees.

## Results

There were 16,329 deliveries to 16,313 women during the 14-month Newhints trial recruitment period, of which 11,274 (69%) were among women who lived in rural areas (i.e. excluding residents of urban towns). In towns, women lived a median of 0.7 km (IQR 0.4–0.9) to the closest health facility and more than 90% of deliveries took place in a health facility. Therefore the investigation of the influence of distance and quality on facility delivery was restricted to the 11,274 deliveries in 310 villages in rural areas of the seven districts in Brong Ahafo region.

Median distance to the closest delivery facility for rural women was 3.3 km (IQR 0.7-7.6, Table 1). When quality was considered, median distances were much longer: rural women lived a median of 10 km to a facility with high or highest quality routine delivery care, 14 km to an EmOC facility and 24 km to an EmNC facility (Table 1, Supplementary Figure 1).

Most individual-level socio-economic and obstetric variables were strongly associated with facility delivery in bivariable analyses, but the Newhints intervention was not (Table 2).

Overall, 58% of the 11,274 deliveries were in a health facility and there was a strong distance decay: 79% of women living within 1 km of a facility had a facility birth but only 28% of women living more than 10 km from their closest facility (Table 3). In models adjusted for individual-level determinants of facility delivery, women living between 1 km and 5 km of their closest facility had 68% lower odds of delivering in a facility compared to women living within 1 km of a facility, and women living farther than 10 km from a facility had 90% lower odds of delivering in a facility than those within 1 km (Table 3). In adjusted models using distance as a continuous variable, the odds of facility delivery decreased by 44% for each one-step increase in square root-transformed distance (i.e. from 1 km to 4 km to 9 km, etc.).

Facility type and quality of care at the closest delivery facility were crudely associated with facility delivery, but in the multivariable multilevel models adjusting for distance and individual-level confounders, there was no clear trend and few effects remained. There was some evidence of higher facility delivery in women whose closest facility was a maternity home or clinic compared to a health center, but not if the closest facility was a hospital (Table 3). There was no evidence that living close to EmOC or EmNC facilities increased facility delivery, although there was evidence that closest facilities with substandard care on the EmOC dimension were associated with 43% lower odds of facility delivery (Table 3). There was some evidence that routine delivery care was associated with facility use, but in a non-intuitive pattern and there was evidence against a linear trend across categories (Likelihood Ratio Test p = 0.02).

**Figure 1:**
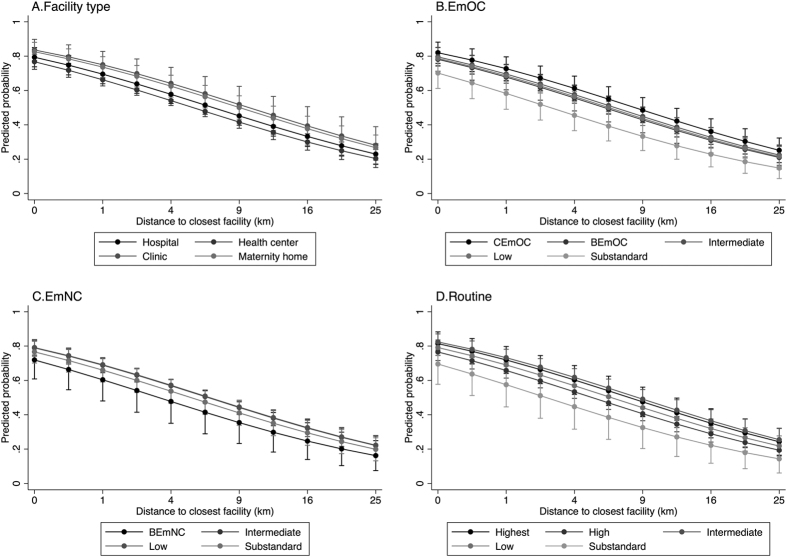
Predicted probability of facility delivery by distance to closest facility, stratified by facility type (**A**) and quality of care (Emergency obstetric care quality (**B**), Emergency neonatal care quality (**C**), Routine care quality (**D**)), based on the models in Table 3.

Figure 1 displays predicted probabilities of facility delivery by distance and quality of closest facility, calculated from the adjusted models in Table 3. Overall, the probability of facility delivery ranged from 78% at a fictive 0 km and 68% at 1 km to 22% at 25 km from a facility, not adjusting for quality. The predicted probabilities stratified by type and quality at the closest facility lie relatively close together with wide confidence intervals, reflecting the lack of evidence that facility delivery is influenced by facility type or quality of care at the closest facility.

There was also no evidence of an association of quality of care at the closest facility with facility delivery when using the clinical vignettes score as a measure for quality. Using the simple vignette quality score, the crude model suggested an association, but there was no evidence to support this in the adjusted model (Supplementary table 1).

When investigating the (combined) effect of distance and quality of care on facility delivery, we found that 64% of women living less than 10 km from an EmOC facility had a facility birth, but only 42% of women living more than 30 km away from such a facility. After adjusting for individual-level confounders in the regression model, however, there was no evidence that distance to EmOC influenced facility delivery. This was also true for distance to CEmOC facilities specifically. Similarly, facility delivery was 70% among women at less than 5 km distance from a closest facility offering good quality routine care, reducing to 52% in those living more than 15 km from such a facility, but there was no evidence for an association in the adjusted regression model. Distances to EmNC were longest and facility delivery was only 32% among women living more than 45 km away. While we found significant associations with facility delivery in the adjusted model, these did not show a meaningful trend. It is difficult to disentangle distance and quality effects using these combined variables; and the reduction in delivery use found for the farthest distance to EmNC may be attributed to distance rather than quality, as women living this far from an EmNC facility were also living far from any facility (Supplementary Table 2).

When investigating the effect of quality of the best facility within a specified distance of the village (again a combined measure of distance and quality, this time keeping distance constant), facility delivery was lowest if no facility was within the specified catchment area (2, 5 or 10 km), but there were again few differences between quality categories in adjusted models. There was some evidence of lower odds of facility delivery when a substandard facility was within the specified catchment area (for EmOC and routine care), but no evidence that facility delivery increased with increasing quality (Supplementary Tables 3–5).

## Discussion

In the study area in Brong Ahafo region, 58% of rural women delivered in a health facility, ranging from almost 80% in women living within 1 km of a facility to less than 30% in women living more than 10 km away. After adjusting for confounders, distance to closest facility was still a strong determinant of facility delivery: the odds of facility delivery decreased by 44% for each one-unit increase in square root-transformed distance from the closest facility. This corresponds to a probability of facility use of 68% for women living 1 km from a health facility and 22% for women living more than 25 km from a facility, keeping other factors constant.

Quality of the closest facility was a determinant of facility delivery insofar as substandard quality on the EmOC dimension decreased use as compared to intermediate quality, but facility delivery was not higher if the closest facility provided EmOC or EmNC. This suggests that a distinction in measured quality at levels above substandard EmOC did not affect facility use for delivery. Facility type affected use in that women whose closest facility was a maternity home were more likely to have a facility delivery compared to those living near a health center. In the study area, all maternity homes are privately owned and staffed by a median of two midwives, whose presence in a village community may encourage facility use. There was also borderline evidence that women whose closest facility was a clinic were more likely to deliver in a facility compared to those living near a health center, but closeness to a hospital did not influence facility use in our rural population.

It has been shown on a national level that a high proportion of Ghanaian women have access to delivery care within a reasonable distance, but that access to EmOC remains poor^24^. We found that almost 85% of rural women in our study area lived within 10 km of a delivery facility but that only 30% lived within 10 km of one capable to provide EmOC. The lower proportion of facility delivery at longer distances to care in our study is consistent with the literature on facility delivery in Ghana and other LMIC in general^9^,^12^. The odds of facility delivery were reduced by 24% on a national level in Ghana for every additional hour of travel time and by 54% for a one-unit increase in log distance^12^. Applying a log transformation to the straight-line distance in our data (instead of the square root transformation chosen because of a better fit) results in a 38% reduction in the odds of facility delivery per unit increase in log distance in fully adjusted models. This suggests that in our study area, distance is less of a barrier to delivery care than in Ghana as a whole and similar to the distance decay reported for Zambia^9^.

We did not find evidence for an association between higher quality of care at the closest facility and facility delivery, except that facility delivery was lower if the closest facility was classified as substandard on the EmOC dimension. The above-mentioned national study in Ghana did not find any effect of emergency obstetric and newborn care (EmONC) quality on use, and the authors hypothesized that this may be due to lack of detail in their quality measure as they categorized facilities into three groups based on the provision of signal functions alone^12^. Another study in Ghana investigated the role of CHPS compounds in increasing facility use and also found that EmONC level of closest facility did not influence use^13^. While an association between EmOC level and facility delivery could be established in Zambia^9^, studies in other settings have also found no association between facility delivery and quality of care using various quality measures, e.g. in Malawi where facility quality was categorized into six levels using staffing and other characteristics^10^.

Our quality classification is arguably quite detailed, incorporating signal functions as well as requirements on staff and their specific skills, opening hours, referral capacity and some tracer items for drugs and equipment^7^. We also assessed quality of routine delivery and postnatal care and newborn emergency care in addition to EmOC, and found no clear trend with these quality dimensions and facility delivery either. It is possible that an even more detailed assessment may be required to evaluate aspects of quality that encourage or discourage use in this context. Unlike the Averting Maternal Death and Disability national-level facility assessments, we did not follow the recommendation in the UN guidelines^1^ and used reported theoretical availability of signal functions, not what was actually provided in the last three months, because we were concerned about putting facilities with low birth loads at a disadvantage. To evaluate in how far our overestimation of facility quality influenced our results, we performed a sensitivity analysis using actual provision of EmOC and EmNC signal functions in the previous six months to categorize facilities, instead of reported theoretical ability to perform these functions. While this did change the categorization of some facilities (three facilities were no longer classified as EmOC, and one no longer as EmNC), the association with facility use for delivery was not substantially different (Supplementary Table 5).

Women’s perception of facility quality is arguably the decisive factor for care-seeking and medical quality of care, no matter how detailed it is measured, can only influence care-seeking in as far as it is perceived as such by women. A literature review of qualitative studies on barriers and facilitators of facility use for delivery found that perception of quality of care provided in facilities was an important determinant of use and varied within the same catchment area, implying that the association between subjective perception of quality and objective measures of quality is not straightforward^25^. A Tanzanian study found that individual characteristics such as education and media exposure, as well as aspects of past and current maternal health care experiences influence perceived quality of delivery care in facilities^26^. Interestingly, they also found that structural facility indicators (availability of drugs, infrastructure and equipment), as well as the number of emergency obstetric services provided at the facility in the last three months did not influence ratings of perceived quality of care^26^. Therefore, if medical quality does not necessarily affect perceived quality, and it is perceived quality that influences care-seeking, this could explain the lack of association with measured medical quality variables in our and other studies.

It can be argued that routine services may have a stronger influence on perceived quality of care as these services are used by more women, whereas emergency services are only used by the minority who experience a complication, and who may have difficulties to judge quality in an emergency. In line with this, the number of postnatal services routinely provided at a facility increased the perception of quality of care in Tanzania^26^. In our study, however, we did not find a clear or convincing association of facility use with measured quality of routine delivery and postnatal care. To investigate non-medical aspects of facility quality, we additionally evaluated patient toilet facilities and whether or not women were able to have a delivery companion, yet did not find any clear association with facility delivery either (data not shown). We were unable to assess other, arguably more important, aspects of non-medical quality, such as interpersonal contact and perceived provider competence, which have been shown to be associated with perceived quality of care^26^.

A strength of this analysis is the use of high-quality population surveillance data. The four-weekly surveillance system was established in the seven districts in Brong Ahafo region in 2000 for a trial of vitamin A supplementation on maternal survival, and large efforts were undertaken to ensure that no pregnancies were missed, even for women living in remote villages^27^. A potential limitation of these data pertains to migration. In this area of Ghana, it is common practice that women leave their village to stay with their natal family during late pregnancy and for delivery, so that their mothers can help with the delivery and postnatal care. Distance to care was determined from an identification code given to women during the surveillance visits. Some women included in the analysis were not seen in the months before delivery and it is possible that just before delivery they were not living at the same place as where they were last seen by the surveillance workers and that their distance to care was thus different from what we estimated. The inclusion of such women may bias the results if migration was differential with respect to distance and delivery location. However, in a sensitivity analysis of a subgroup of women with long delays between visits, there was no evidence of an association with distance to care or facility delivery, suggesting that any potential misclassification was non-differential, which would result in an underestimation of the effect.

While it is commonly upheld that straight-line distance is a poor proxy for access to care, we have previously shown that it can be used as a sufficient measure of access in this area^22^. Nevertheless, we compared several more sophisticated access measures in a sensitivity analysis, including road distance and raster travel time from both villages and compounds, and found very similar results to those presented using straight-line distance (not shown).

## Conclusion

Distance is an important obstacle to facility delivery in rural Ghana, such that women at further distances either do not decide to seek care or are unable to identify and access a facility before delivery. Measured quality of delivery care at the closest health facility, however, did not seem to play an important role in facility use for delivery in this region of Ghana, except for a reduction in facility delivery when the quality of care provided at the closest facility was substandard on the EmOC dimension. This could be interpreted to mean that women do not differentiate between the higher levels of care, or that if they do perceive differences, this does not influence their decision to deliver in a facility. While we found no evidence that quality at the closest facility influenced whether or not women delivered in a facility in general, this does not preclude the influence of quality on the choice of a specific facility for delivery. It is possible that quality influences whether or not women seek care at their closest facility or choose to travel to a different facility farther away, a phenomenon known as bypassing^28^, which warrants further investigation in this context. Therefore, absence of a clear effect of quality on facility use does not imply that we should increase geographic coverage with facilities unable to provide high quality routine and emergency care. While better access to care regardless of quality seems to encourage women to use facilities for delivery, the limited content of care women receive at low quality facilities cannot be expected to reduce maternal and neonatal mortality.

## Additional Information

**How to cite this article**: Nesbitt, R. C. *et al*. The influence of distance and quality of care on place of delivery in rural Ghana. *Sci. Rep.* **6**, 30291; doi: 10.1038/srep30291 (2016).

## Supplementary Material

Supplementary Information

## Figures and Tables

**Table 1:** Distance to delivery facilities considering quality dimensions, n = 11,274 deliveries.

Distance to:	Number of facilities n (%)	Median distance in km (IQR)	Range in km (min-max)
Any delivery facility	64 (100%)	3.3 (0.7–7.6)	0.03–23.4
Hospital	11 (17%)	13.9 (8.6–20.5)	0.47–84.0
Facility with good routine care*	34 (53%)	10.3 (5.2–14.6)	0.02–38.5
EmOC facility**	12 (19%)	14.1 (8.3–21.5)	0.80–84.0
EmNC facility***	5 (8%)	23.6 (14.3–37.3)	0.21–119.1

*Good routine care = high or highest on the quality of care categorization, i.e. providing ≥8/12 signal functions; ≥3 health professionals conducting deliveries (≥1 of whom midwives) employed at the facility. **EmOC = basic or comprehensive emergency obstetric care i.e. providing all 6 basic signal functions (or all except assisted vaginal delivery); ≥3 health professionals (≥1 of whom is a midwife) managing obstetric complications and ≥1 present during the visit.

***EmNC = basic or comprehensive emergency newborn care i.e. providing ≥5 signal functions (or all except dexamethasone to mother for premature labour); ≥3 health professionals managing sick newborns and ≥1 present during the visit.

See ref. 7 for details on the quality classification.

**Table 2:** Crude associations of individual determinants with facility delivery.

		Deliveries		Facility deliveries		Chi-square p-value	Odds Ratio^1^ (95% CI), p-value
Individual covariate		n	%	n	%		
**Age (years)**	15–19	1381	12.4	910	65.9	<0.001	1.0
	20–29	5791	51.9	3269	56.5		0.62 (0.53–0.71)
	30+	3979	35.7	2261	56.8		0.61 (0.53–0.71)
	Total	11151	100	6440	57.8		<0.001
**Parity**	0	2412	21.6	1714	71.1	<0.001	1.0
	1–2	3969	35.6	2278	57.4		0.46 (0.40–0.52)
	3–4	2778	24.9	1474	53.1		0.41 (0.36–0.47)
	5+	1992	17.9	974	48.9		0.40 (0.35–0.47)
	Total	11151	100	6440	57.8		<0.001
**Previous stillbirth**	None	10291	92.3	5922	57.6	0.31	1.0
	One	695	6.2	419	60.3		1.20 (1.00–1.44)
	Two or more	165	1.5	99	60.0		1.22 (0.85–1.77)
	Total	11151	100	6440	57.8		0.10
**Multiple birth**	Single	10924	98.0	6266	57.4	<0.001	1.0
	Multiple	227	2.0	174	76.7		3.24 (2.28–4.60)
	Total	11151	100	6440	57.8		<0.001
**Marital status**	Married	6052	54.3	3185	52.6	<0.001	1.0
	Cohabitating	3942	35.4	2512	63.7		1.32 (1. 19–1.49)
	Single	1156	10.4	742	64.2		1.61 (1.37–1.89)
	Total	11151	100	6439	57.8		<0.001
**Wealth quintile**	Poorest	3177	28.5	1254	39.4	<0.001	1.0
	Second	2944	26.5	1497	50.9		1.60 (1.41–1.81)
	Middle	2515	22.6	1671	66.4		2.40 (2.09–2.75)
	Fourth	1784	16.0	1394	78.1		3.43 (2.90–4.06)
	Least poor	709	6.4	614	86.6		5.08 (3.90–6.60)
	Total	11129	100	6430	57.8		<0.001
**Education**	None	4274	38.3	1781	41.7	<0.001	1.0
	Primary	2433	21.8	1422	58.5		1.76 (1.56–1.98)
	Middle	3967	35.6	2831	71.4		2.68 (2.40–3.00)
	College	473	4.2	403	85.2		5.37 (4.02–7.16)
	Total	11147	100	6437	57.8		<0.001
**Occupation**	Professional	1024	9.2	761	74.3	<0.001	1.0
	Unskilled	7073	63.4	3863	54.6		0.51 (0.43–0.60)
	Unemployed	3054	27.4	1816	59.5		0.59 (0.49–0.70)
	Total	11151	100	6440	57.8		<0.001
**Religion**	Catholic	2868	25.7	1527	56.9	<0.001	1.0
	Protestant	5096	45.7	3417	67.1		1.41 (1.26–1.59)
	Muslim	2264	20.3	1037	45.8		0.68 (0.59–0.79)
	Traditional	1105	9.9	459	41.5		0.65 (0.55–0.77)
	Total	11151	100	6440	57.8		<0.001
**Ethnicity**	Akan	4598	41.2	3287	71.5	<0.001	1.0
	Other	6553	58.8	3153	48.1		0.45 (0.39–0.50)
	Total	11151	100	6440	57.8		<0.001
**Health insurance**	Yes	9368	84.0	5667	60.5	<0.001	1.0
	No	1779	16.0	773	43.5		0.46 (0.40–0.52)
	Total	11147	100	6440	57.8		<0.001
**Newhints intervention**	Control	5441	48.8	3017	55.5	<0.001	1.0
	Intervention	5710	51.2	3423	60.0		1.09 (0.83–1.44)
	Total	11151	100	6440	57.8		0.55

^1^Odds ratios were calculated using multilevel logistic regression models with random intercepts for village.

**Table 3:** Association of distance, facility type and quality of care at closest facility with facility delivery.

	Deliveries n (%)	Facility deliveries, n (%)^2^	Crude OR (95% CI), p-value^3^, n = 11,274	Adjusted^4^ OR (95% CI), p-value^3^, n = 11,129
**Distance to closest delivery facility (of any level)**				
<1 km	3890 (34.5)	3060 (78.7)	1.0	1.0
1–5 km	2876 (25.5)	1736 (60.4)	0.32 (0.23–0.45), <0.001	0.32 (0.23–0.44), <0.001
5–10 km	2786 (24.7)	1248 (44.8)	0.20 (0.14–0.28), <0.001	0.27 (0.20–0.38), <0.001
>10 km	1722 (15.3)	479 (27.8)	0.10 (0.07–0.15), <0.001	0.16 (0.11–0.24), <0.001
**Distance to closest delivery facility (of any level; distance as a continuous variable)**				
Sqrt-distance^1^			0.47 (0.42–0.53), <0.001	0.56 (0.50–0.63), <0.001
**Distance to closest delivery facility + type of closest facility**				
Sqrt-distance^1^			0.47 (0.42–0.53), <0.001	0.56 (0.50–0.63), <0.001
Hospital	1657 (14.7)	933 (56.3)	1.26 (0.95–1.67), 0.11	1.18 (0.90–1.57), 0.24
Health center	7650 (67.9)	4389 (57.4)	1.0	1.0
Clinic	687 (6.1)	475 (69.1)	1.26 (0.77–2.06), 0.35	1.61 (0.99–2.60), 0.05
Maternity home	1280 (11.4)	726 (56.7)	1.42 (1.01–2.00), 0.04	1.49 (1.06–2.08), 0.02
**Distance to closest delivery facility + EmOC quality of closest facility**				
Sqrt-distance^1^			0.46 (0.42–0.52), <0.001	0.56 (0.50–0.62), <0.001
CEmOC	648 (5.8)	379 (58.5)	1.32 (0.89–1.97), 0.17	1.19 (0.79–1.78), 0.41
BEmOC	1.52 (9.3)	550 (52.3)	0.98 (0.65–1.48), 0.94	0.91 (0.60–1.38), 0.67
Intermediate	2785 (24.7)	1592 (57.2)	1.0	1.0
Low	5876 (52.1)	3682 (62.7)	0.95 (0.72–1.24), 0.70	0.94 (0.71–1.25), 0.71
Substandard	913 (8.1)	320 (35.1)	0.43 (0.27–0.68), <0.001	0.57 (0.35–0.92), 0.02
**Distance to closest delivery facility + EmNC quality of closest facility**				
Sqrt-distance^1^			0.47 (0.42–0.52), <0.001	0.56 (0.50–0.62), <0.001
CEmNC	0	0		
BEmNC	298 (2.6)	204 (68.5)	0.71 (0.39–1.31), 0.28	0.65 (0.35–1.21), 0.18
Intermediate	3967 (35.2)	2329 (58.7)	1.0	1.0
Low	5464 (48.5)	3214 (58.8)	0.86 (0.68–1.08), 0.20	1.01 (0.80–1.28), 0.94
Substandard	1545 (13.7)	776 (50.2)	0.67 (0.46–0.97), 0.03	0.86 (0.59–1.25), 0.42
**Distance to closest delivery facility + routine delivery care quality of closest facility**				
Sqrt-distance^1^			0.46 (0.41–0.51), <0.001	0.55 (0.50–0.62), <0.001
Highest	1087 (9.6)	667 (61.4)	1.13 (0.72–1.76), 0.60	0.93 (0.59–1.48), 0.76
High	3760 (33.4)	2007 (53.4)	0.73 (0.54–0.98), 0.03	0.67 (0.50–0.90), 0.01
Intermediate	2482 (22.0)	1697 (68.4)	1.0	1.0
Low	3584 (31.8)	1951 (54.4)	0.70 (0.52–0.95), 0.02	0.79 (0.58–1.08), 0.14
Substandard	361 (3.2)	201 (55.7)	0.31 (0.16–0.59), <0.001	0.45 (0.23–0.87), 0.02

Middle categories (health center, intermediate quality) were chosen as reference group to increase power.

^1^Straight-line distance was square root transformed, e.g., 4 km–> √4 = 2; 9 km–> √9 = 3. ORs for facility delivery reflect the change per one unit of the transformed variable, i.e. from 1 km to 4 km to 9 km, etc.

^2^Chi square tests for % facility delivery across categories, all variables: p < 0.001.

^3^Wald test p-values for each level of categorical variables.

^4^Covariates in adjusted models include: age, parity, previous stillbirth, multiple birth, marital status, wealth quintile, education, occupation, religion, ethnicity, health insurance and Newhints intervention zone.
