# Supplementary material for: The influence of distance and quality of care on place of delivery in rural Ghana
Source: Sci Rep. 2016 Aug 10;6:30291. doi: 10.1038/srep30291 (PMC4978958; doi:10.1038/srep30291)
Supplement: Supplementary Information [file srep30291-s1.pdf]

Title: The influence of distance and quality of care on place of delivery in rural Ghana

Authors: Robin C. Nesbitt, Terhi J. Lohela, Seyi Soremekun, Linda Vesel, Alexander Manu, Eunice Okyere, Chris Grundy, Seeba Amenga-Etego, Seth Owusu-Agyei, Betty R. Kirkwood, Sabine Gabrysch

Author Affiliations:

Robin C Nesbitt, MSc DrScHum *

Epidemiology and Biostatistics Unit, Institute of Public Health, Heidelberg University, Heidelberg, Germany

*Corresponding author. Email: robin.nesbitt@uni-heidelberg.de

Terhi J Lohela, MD MScIH

Department of Public Health, University of Helsinki, Helsinki, Finland

Email: terhi.lohela@helsinki.fi

Seyi Soremekun, MSc PhD

Maternal & Child Health Intervention Research Group, Faculty of Epidemiology and Population Health, London School of Hygiene and Tropical Medicine, London, UK

Email: seyi.soremekun@lshtm.ac.uk

Linda Vesel, MPH PhD

Innovations for Maternal, Newborn and Child Health Initiative, Concern Worldwide US, New York, USA

Email: linda.vesel@gmail.com

Alexander Manu, MBChB MSc PhD

Kintampo Health Research Center, Ghana Health Service, Kintampo, Ghana; Maternal & Child Health Intervention Research Group, Faculty of Epidemiology and Population Health, London School of Hygiene and Tropical Medicine, London, UK

Email: makmanu128@gmail.com

Eunice Okyere, MScIH

Flinders University, Department of Public Health, Adelaide, South Australia.

Email: euniceadoma@yahoo.co.uk

Chris Grundy, MSc

Department of Infectious Disease Epidemiology, Faculty of Epidemiology and Population Health, London School of Hygiene and Tropical Medicine, London, UK

Email: chris.grundy@lshtm.ac.uk

Seeba Amenga-Etego, MSc

Kintampo Health Research Center, Ghana Health Service, Kintampo, Ghana

Email: seeba.ae@kintampo-hrc.org

Seth Owusu-Agyei, PhD

Director, Kintampo Health Research Center, Ghana Health Service, Kintampo, Ghana

Email: seth.owusu-agyei@kintampo-hrc.org

Betty R Kirkwood, MA MSc DIC FFPH FMedSci

Maternal & Child Health Intervention Research Group, Faculty of Epidemiology and Population Health, London School of Hygiene and Tropical Medicine, London, UK

Email: Betty.Kirkwood@lshtm.ac.uk

Sabine Gabrysch, MD PhD

Unit of Epidemiology and Biostatistics, Institute of Public Health, Heidelberg University, Heidelberg, Germany.

Email: sabine.gabrysch@uni-heidelberg.de

Supplementary tables and figures

Supplementary table 1. Association between facility delivery and quality of care using alternative quality measures, n=11,274.

	**Deliveries n (%)**		**Facility delivery n (%)**[2]		**Crude OR (95% CI), p-value**[3] **n= 11,274**			**Adjusted**[4] **OR (95% CI), p-value**[3] **n= 11,129**		
**Distance to closest delivery facility and vignette score**[5] **of the closest facility**										
Sqrt distance[1]					0.47	0.42-0.53	<0.001	0.56	0.50-0.63	<0.001
Low (<50%)	2485	22.0	1287	51.8	1.0			1.0		
Moderate (50-75%)	8789	78.0	5236	59.6	1.02	0.99-1.05	0.14	1.01	0.98-1.04	0.49
High (>75%)	0	0								
**Distance to closest delivery facility and simple quality score**[6] **of the closest facility**										
Sqrt distance[1]					0.46	0.41-0.51	<0.001	0.55	0.49-62	<0.001
Low (<50%)	2982	26.5	1561	52.4	1.0			1.0		
Moderate (50-75%)	7400	92.1	4425	59.8	1.38	1.07-1.79	0.014	1.19	0.92-1.54	0.19
High (>75%)	892	7.9	537	60.2	1.77	1.22-2.57	0.003	1.38	0.94-2.01	0.10

Notes:

[1]Straight-line distance was square root transformed, e.g., 4km --> √4 = 2, 9km --> √9 = 3. ORs for facility delivery reflect the change per one unit of the transformed variable, i.e. from 1km to 4km to 9km, etc.

[2] Chi square tests for % facility delivery across categories, both variables: p<0.001

[3] Wald test p-values for each level of categorical variables

[4] Covariates in adjusted models include: age, parity, previous stillbirth, multiple birth, marital status, wealth quintile, education, occupation, religion, ethnicity, health insurance and Newhints intervention zone.

[5]For details on the vignette score, please see reference 24.

[6]The simple quality score was calculated using one point per signal function, one point per health professional (up to 3), one point per doctor conducting cesarean section (up to 3), to a maximum total of 32 points.

Supplementary table 2. Association between facility delivery and distance to delivery care of a certain quality, n=11,274.

	Deliveries n (%)		Facility delivery n (%)[1]		Crude OR (95% CI), p-value[2] n= 11,274			Adjusted OR[3] (95% CI), p-value[2] n= 11,129		
**Distance to closest EmOC facility (BEmOC or CEmOC)**										
< 10 km	3420	30.3	2198	64.3	1.0			1.0		
10 - 20 km	4657	41.3	2588	55.6	0.70	0.51-0.98	0.04	0.85	0.63-1.15	0.29
20 - 30 km	1915	17.0	1199	62.6	0.76	0.50-1.14	0.18	1.12	0.77-1.62	0.57
> 30 km	1282	11.4	538	42.0	0.62	0.37-1.04	0.07	0.87	0.54-1.39	0.56
**Distance to closest CEmOC facility**										
< 10 km	2667	23.7	1795	67.3	1.0			1.0		
10 - 20 km	4388	38.9	2483	56.6	0.72	0.51-1.03	0.07	0.87	0.63-1.20	0.39
20 - 30 km	2937	26.1	1707	58.1	0.67	0.46-0.98	0.04	0.97	0.68-1.38	0.85
> 30 km	1282	11.4	538	42.0	0.59	0.35-1.01	0.05	0.86	0.53-1.39	0.53
**Distance to closest facility with good routine care (High or Highest)**										
< 5 km	2743	24.3	1917	69.9	1.0			1.0		
5 - 10 km	2844	25.2	1567	55.1	0.60	0.41-0.89	0.01	0.81	0.57-1.16	0.25
10 - 15 km	3111	27.6	1713	55.1	0.51	0.34-0.77	0.001	0.80	0.55-1.16	0.24
> 15 km	2576	22.9	1326	51.5	0.55	0.37-0.83	0.004	0.92	0.63-1.33	0.65
**Distance to closest EmNC facility (BEmNC or CEmNC)**										
<15 km	3288	29.2	2089	63.3	1.0			1.0		
15 - 30 km	3582	31.8	2175	60.2	0.90	0.64-1.25	0.51	1.10	0.81-1.50	0.55
30 - 45 km	2629	23.3	1693	64.4	1.07	0.73-1.58	0.72	1.51	1.05-2.16	0.03
> 45	1775	15.7	566	31.9	0.35	0.23-0.52	<0.001	0.60	0.41-0.88	0.01
**Distance to closest hospital**										
< 10 km	3592	31.9	2361	65.7	1.0			1.0		
10 - 15 km	2940	26.1	1632	55.5	0.72	0.50-1.03	0.08	0.83	0.59-1.15	0.26
15 - 25 km	3346	29.7	2005	59.9	0.73	0.51-1.04	0.08	0.99	0.71-1.36	0.93
> 25 km	1396	12.4	525	37.6	0.50	0.32-0.79	0.003	0.74	0.49-1.12	0.16

Notes:

[1] Chi square tests for % facility delivery across categories, both variables: p<0.001

[2] Wald test p-values for each level of categorical variables

[3] Covariates in adjusted models include: age, parity, previous stillbirth, multiple birth, marital status, wealth quintile, education, occupation, religion, ethnicity, health insurance and Newhints intervention zone.

Supplementary table 3. Association between facility delivery and EmOC quality of best facility within a certain distance.

	Deliveries n (%)		Facility delivery n (%)[1]		Crude OR (95% CI), p-value[2] n= 11,274			Adjusted[3] OR (95% CI), p-value[2] n= 11,129		
**EmOC quality provided at best facility within 2 km**										
None	6477	57.5	2794	43.1	0.16	0.084-0.30	<0.001	0.25	0.14-0.46	<0.001
Substandard	445	4.0	234	52.6	0.20	0.079-0.52	<0.001	0.37	0.15-0.89	<0.001
Low	2838	25.2	2275	80.2	0.83	0.42-1.66	0.60	1.03	0.54-1.97	0.93
Intermediate	1244	11.0	996	80.1	1			1		
BEmOC	195	1.7	156	80.0	0.87	0.26-2.84	0.81	1.18	0.38-3.64	0.78
CEmOC	75	0.7	68	90.7	2.10	0.44-9.99	0.35	1.45	0.33-6.38	0.62
**EmOC quality provided at best facility within 5 km**										
None	4508	40.0	1727	38.3	0.30	0.18-0.50	<0.001	0.48	0.30-0.78	0.0031
Substandard	454	4.0	234	51.5	0.36	0.15-0.86	0.021	0.60	0.26-1.41	0.24
Low	3862	34.3	2741	71.0	0.89	0.53-1.50	0.67	1.01	0.61-1.68	0.96
Intermediate	1604	14.2	1202	74.9	1			1		
BEmOC	285	2.5	188	66.0	0.54	0.22-1.32	0.18	0.75	0.31-1.78	0.51
CEmOC	561	5.0	431	76.8	1.37	0.70-2.68	0.36	1.22	0.63-2.37	0.55
**EmOC quality provided at best facility within 10 km**										
None	1722	15.3	479	27.8	0.30	0.19-0.48	<0.001	0.41	0.26-0.63	<0.001
Substandard	285	2.5	79	27.7	0.30	0.13-0.71	0.0059	0.37	0.16-0.82	0.015
Low	4425	39.3	2821	63.8	0.98	0.65-1.48	0.91	1.02	0.69-1.50	0.93
Intermediate	1422	12.6	946	66.5	1			1		
BEmOC	753	6.7	403	53.5	0.82	0.43-1.53	0.53	0.79	0.44-1.42	0.42
CEmOC	2667	23.7	1795	67.3	1.04	0.67-1.61	0.85	0.88	0.58-1.32	0.53

Notes:

[1] Chi square tests for % facility delivery across categories, both variables: p<0.001

[2] Wald test p-values for each level of categorical variables

[3] Covariates in adjusted models include: age, parity, previous stillbirth, multiple birth, marital status, wealth quintile, education, occupation, religion, ethnicity, health insurance and Newhints intervention zone.

Supplementary table 4. Association between facility delivery and EmNC quality of the best facility within a certain distance.

	Deliveries n (%)		Facility delivery n (%)[2]		Crude OR (95% CI), p-value[3] n= 11,274			Adjusted[4] OR (95% CI), p-value[3] n= 11,129		
**EmNC quality provided at best facility within 2 km**										
None	6477	57.5	2794	43.1	0.14	0.088-0.24	<0.001	0.21	0.13-0.34	<0.001
Substandard	687	6.1	474	69.0	0.39	0.18-0.87	0.021	0.59	0.28-1.23	0.16
Low	2636	23.4	2064	78.3	0.72	0.40-1.28	0.26	0.79	0.46-1.36	0.40
Intermediate	1387	12.3	1131	81.5	1			1		
BEmOC	87	0.77	60	69.0	0.31	0.10-0.96	0.042	0.37	0.13-1.08	0.069
CEmOC[1]										
**EmNC quality provided at best facility within 5 km**										
None	4508	40.0	1727	38.3	0.35	0.25-0.49	<0.001	0.54	0.39-0.76	<0.001
Substandard	700	6.2	461	65.9	0.71	0.34-1.48	0.37	1.24	0.62-2.50	0.55
Low	3370	29.9	2419	71.8	1.12	0.75-1.67	0.57	1.28	0.87-1.87	0.21
Intermediate	2344	20.8	1670	71.3	1			1		
BEmOC	352	3.1	246	69.9	0.87	0.44-1.71	0.69	0.77	0.40-1.49	0.44
CEmOC										
**EmNC quality provided at best facility within 10 km**										
None	1722	15.3	479	27.8	0.32	0.22-0.47	<0.001	0.46	0.32-0.65	<0.001
Substandard	600	5.3	293	48.8	0.48	0.24-0.95	0.035	0.61	0.32-1.16	0.13
Low	2965	26.3	1779	60.0	0.91	0.66-1.26	0.58	1.18	0.86-1.61	0.31
Intermediate	3998	35.5	2557	64.0	1			1		
BEmOC	1989	17.6	1415	71.1	1.41	0.97-2.06	0.074	1.06	0.74-1.52	0.75
CEmOC										

Notes:

[1]CEmOC facility row is blank because no CEmOC facilities were within 10 km of a village (closest is 20.7 km).

[2] Chi square tests for % facility delivery across categories, both variables: p<0.001

[3] Wald test p-values for each level of categorical variables

[4]Covariates in adjusted models include: age, parity, previous stillbirth, multiple birth, marital status, wealth quintile, education, occupation, religion, ethnicity, health insurance and Newhints intervention zone.

Supplementary table 5. Association between facility delivery and routine care quality of the best facility within a certain distance.

	**Deliveries n (%)**		**Facility delivery n (%)[1]**		**Crude OR (95% CI), p-value[2] n= 11,274**			**Adjusted OR (95% CI), p-value[3] n= 11,129**		
**Routine delivery care quality provided at best facility within 2 km**										
None	6477	57.5	2794	43.1	0.14	0.078-0.25	<0.001	0.21	0.12-0.36	<0.001
Substandard	304	2.7	183	60.2	0.22	0.079-0.64	0.0052	0.34	0.13-0.91	0.031
Low	1652	14.7	1203	72.8	0.50	0.26-0.99	0.045	0.64	0.34-1.20	0.17
Intermediate	1388	12.3	1137	81.9	1			1		
High	1059	9.4	873	82.4	0.93	0.45-1.94	0.85	1.05	0.53-2.10	0.89
Highest	394	57.5	333	84.5	1.43	0.35-5.80	0.61	1.04	0.29-3.81	0.95
**Routine delivery care quality provided at best facility within 5 km**										
None	4508	40.0	1727	38.3	0.17	0.10-0.28	<0.001	0.27	0.17-0.45	<0.001
Substandard	313	2.8	183	58.5	0.22	0.083-0.60	0.0031	0.36	0.14-0.93	0.036
Low	2008	17.8	1346	67.0	0.51	0.29-0.89	0.018	0.66	0.39-1.14	0.14
Intermediate	1702	15.1	1350	79.3	1			1		
High	1967	17.5	1297	65.9	0.37	0.21-0.64	<0.001	0.39	0.23-0.67	<0.001
Highest	776	6.9	620	79.9	0.88	0.43-1.82	0.73	0.71	0.35-1.44	0.34
**Routine delivery care quality provided at best facility within 10 km**										
None	1722	15.3	479	27.8	0.28	0.17-0.44	<0.001	0.36	0.23-0.56	<0.001
Substandard	125	1.1	44	35.2	0.32	0.12-0.87	0.026	0.38	0.15-0.97	0.043
Low	1917	17.0	1167	60.9	0.98	0.61-1.58	0.94	1.14	0.73-1.78	0.56
Intermediate	1923	17.1	1349	70.2	1			1		
High	2840	25.2	1640	57.8	0.71	0.46-1.09	0.12	0.67	0.45-1.00	0.048
Highest	2747	24.4	1844	67.1	1.00	0.65-1.54	0.99	0.80	0.53-1.19	0.27

Notes:

[1] Chi square tests for % facility delivery across categories, both variables: p<0.001

[2] Wald test p-values for each level of categorical variables

[3]Covariates in adjusted models include: age, parity, previous stillbirth, multiple birth, marital status, wealth quintile, education, occupation, religion, ethnicity, health insurance and Newhints intervention zone.

Supplementary table 6. Association between facility delivery and distance to care and emergency care quality of the closest facility, using actual provision of signal functions in the previous six months.

	Deliveries n (%)	Facility delivery n (%)[2]	Crude OR (95% CI), p-value[3] n= 11,274	Adjusted[4] OR (95% CI), p-value[3] n= 11,129
**Distance to closest delivery facility + EmOC quality of closest facility**				
Sqrt distance[1]			0.47 (0.42-0.52), <0.001	0.56 (0.50-0.63), <0.001
CEmOC	577 (5.1)	329 (57.0)	1.0	1.0
BEmOC	730 (6.5)	351 (48.1)	0.64 (0.40-1.11), 0.12	0.73 (0.42-1.27), 0.26
Intermediate	239 (2.1)	182 (76.2)	2.10 (0.99-4.46), 0.05	1.76 (0.83-3.77), 0.14
Low	4,702 (41.7)	2,873 (61.1)	0.83 (0.56-1.25), 0.38	0.97 (0.65-1.46), 0.89
Substandard	5,026 (44.6)	2,788 (55.5)	0.68 (0.46-1.02), 0.06	0.79 (0.53-1.19), 0.26
**Distance to closest delivery facility + EmNC quality of closest facility**				
Sqrt distance[1]			0.46 (0.41-0.52), <0.001	0.55 (0.49-0.62), <0.001
CEmNC	0	0		
BEmNC	168 (1.5)	132 (78.6)	1.0	1.0
Intermediate	2,816 (25.0)	1,605 (57.0)	0.37 (0.13-1.02), 0.06	0.67 (0.24-1.89), 0.45
Low	3,646 (32.3)	2,147 (58.9)	0.27 (0.099-0.76), 0.01	0.58 (0.21-1.62), 0.30
Substandard	4,644 (41.2)	2,639 (56.8)	0.24 (0.90-0.68), 0.007	0.55 (0.20-1.53), 0.04

Notes:

[1]Straight-line distance was square root transformed, e.g., 4km --> √4 = 2, 9km --> √9 = 3. ORs for facility delivery reflect the change per one unit of the transformed variable, i.e. from 1km to 4km to 9km, etc.

[2] Chi square tests for % facility delivery across categories, both variables: p<0.001

[3] Wald test p-values for each level of categorical variables

[4] Covariates in adjusted models include: age, parity, previous stillbirth, multiple birth, marital status, wealth quintile, education, occupation, religion, ethnicity, health insurance and Newhints intervention zone.

Supplementary Figure 1. Distances to closest delivery facility and to closest facility providing good quality routine care, emergency obstetric care and emergency newborn care for 11,274 rural deliveries.

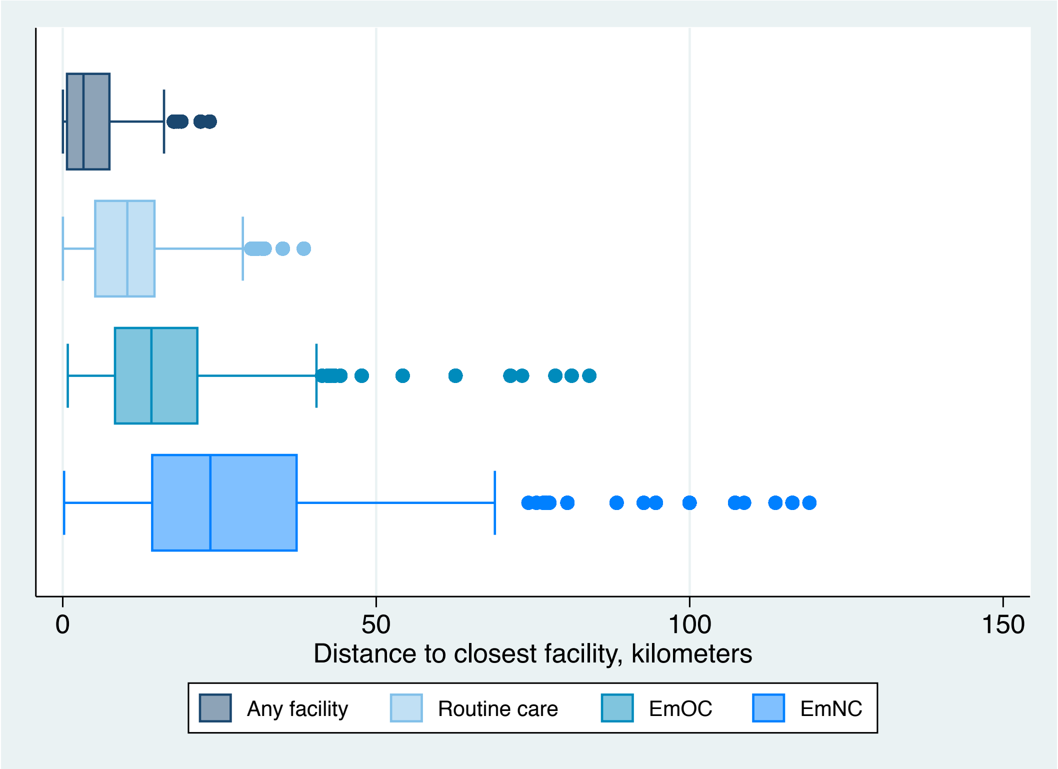

REFERENCES:

23. Lohela, T.J., et al., *Competence of health workers in emergency obstetric care: An assessment using clinical vignettes in Brong Ahafo region, Ghana.* Under Review BMJ Open, 2016.
